# Sex differences in cerebrovascular function across an aerobic exercise intervention in older adults using MRI: Results from the Brain in Motion study

**DOI:** 10.14814/phy2.70880

**Published:** 2026-04-17

**Authors:** Alison M. H. Donald, Rebecca J. Williams, Andrew E. Beaudin, Erin L. Mazerolle, Brandy L. Callahan, G. Bruce Pike, Marc J. Poulin

**Affiliations:** ^1^ Department of Physiology and Pharmacology, Cumming School of Medicine University of Calgary Calgary Alberta Canada; ^2^ Hotchkiss Brain Institute, Cumming School of Medicine University of Calgary Calgary Alberta Canada; ^3^ School of Science and Technology University of New England Armidale New South Wales Australia; ^4^ Department of Clinical Neurosciences, Cumming School of Medicine University of Calgary Calgary Alberta Canada; ^5^ Department of Psychology, Faculty of Arts, Faculty of Science St. Francis Xavier University Antigonish Nova Scotia Canada; ^6^ Department of Computer Science, Faculty of Science St. Francis Xavier University Antigonish Nova Scotia Canada; ^7^ Department of Psychology, Faculty of Arts University of Calgary Calgary Alberta Canada; ^8^ Department of Radiology, Cumming School of Medicine University of Calgary Calgary Alberta Canada; ^9^ Faculty of Kinesiology University of Calgary Calgary Alberta Canada; ^10^ O'Brien Institute for Public Health, Cumming School of Medicine University of Calgary Calgary Alberta Canada; ^11^ Libin Cardiovascular Institute of Alberta, Cumming School of Medicine University of Calgary Calgary Alberta Canada; ^12^ Brenda Strafford Foundation Chair in Alzheimer Research Calgary Alberta Canada

**Keywords:** cardiorespiratory fitness, cerebral blood flow, cerebrovascular reactivity, magnetic resonance imaging, sex differences

## Abstract

Sex differences in cerebral blood flow (CBF) and cerebrovascular reactivity (CVR) with aging, and across an aerobic exercise training intervention are poorly understood. The primary aim of this study was to investigate sex differences in cerebrovascular function in brain regions involved in verbal fluency, across an aerobic exercise intervention. CBF and CVR were assessed in 27 Brain in Motion study participants before, and after a 6‐month aerobic exercise intervention. Resting CBF was measured using arterial spin labeling (ASL) magnetic resonance imaging (MRI) while CVR was quantified using blood oxygen level dependent (BOLD) MRI and a hypercapnic challenge. Compared to pre‐intervention values, $\dot{V}O_{2}\text{peak}$ increased (*p* < 0.001) while whole brain cerebrovascular measures were unchanged (all *p* > 0.05). There was no effect of the intervention or sex on CVR in the hippocampus, insula or left pars triangularis. There was a sex and intervention interaction in hippocampal, insula, and left pars triangularis blood flow (*p* < 0.05). These findings provide evidence of sex differences in regions of the brain involved in verbal fluency in response to an aerobic exercise intervention.

## Graphical abstract

Females and males differ in their cerebral blood flow changes across an aerobic exercise intervention in the regions of the hippocampi, insula, and left pars triangulars.

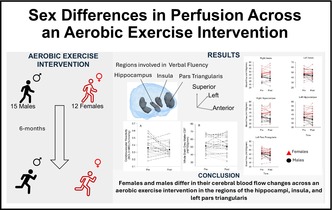

## 1 INTRODUCTION

Sedentary behavior is a major modifiable risk factor for dementia and is directly related to vascular brain health (Livingston et al., 2020). Studies targeting the cerebrovasculature as a modifiable factor contributing to delaying and/or preventing mild cognitive impairment and dementia show promising results (Brown et al., 2010; Eskes et al., 2010; Guadagni et al., 2020; Ngandu et al., 2015). Common measures of cerebrovascular health include resting cerebral blood flow (CBF) and cerebrovascular reactivity (CVR). CVR refers to the ability of the brain to increase CBF in response to vasoactive stimuli (Chen, 2018; Williams et al., 2023) and is typically expressed as a change in CBF, normalized for the change in vasoactive stimulus.

The Brain in Motion (BIM) study investigated the impact of a 6‐month aerobic exercise training program on cognitive function in healthy, sedentary older adults (Tyndall et al., 2013). Following the exercise intervention, verbal memory and verbal fluency showed the greatest improvement (Guadagni et al., 2020). We therefore wanted to investigate cerebrovascular health in brain regions associated with these cognitive domains. To study regional perfusion and CVR in the context of verbal fluency, we looked at the left pars triangularis, a region of the inferior frontal gyrus known as Broca's area (Elmer, 2016), as well as the hippocampus and insula. Importantly, the left pars triangularis has been found to play a primary role in the comprehension and production of language and is also a region necessary for semantic and phonological fluency (Ishkhanyan et al., 2020; Schmidt et al., 2019). However, it remains unclear whether these findings generalize to all older adults, or whether sex differences need to be considered when evaluating verbal memory changes following an exercise intervention. This warrants investigation due to evidence suggesting sex differences in verbal ability and processing (Xu et al., 2020).

We investigated the hippocampus and the insula as they are implicated in cognitive decline and the evolution of mild cognitive impairment (MCI) and dementia (Carmeli et al., 2013; Duan et al., 2021). Exploring these relationships is important to advance our understanding of the impact of exercise in older adults and the utility of CVR as a biomarker for cerebrovascular health, as both advanced aging and sedentary behavior contribute to a greater risk of cognitive decline.

While previous research has highlighted that cognitive changes are evident following an exercise intervention (particularly global cognitive scores (Han et al., 2023)), the extent to which changes are due to sex differences is unclear. Furthermore, there is a lack of research evaluating CVR longitudinally across exercise interventions in older adults, using MRI. A clear age‐related decline in CVR has yet to be established (Claassen et al., 2021). The novel contribution of this study pertains to sex differences in cerebrovascular outcomes within brain regions implicated in verbal fluency across a 6‐month aerobic exercise intervention using MRI. Findings will enhance our knowledge of sex differences and brain region‐specific changes in cerebrovascular regulation and provide a better understanding of the role of exercise on brain health.

Thus, the present study had three research aims. First, we explored sex differences in the impact of an aerobic exercise training program on CBF and CVR in the left pars triangularis, hippocampus, and insular cortex. Second, we investigated global CBF and CVR before and after a 6‐month aerobic exercise intervention. Finally, we investigated the relationship between the change in cognitive test scores and the change in cerebrovascular outcomes across the intervention.

We hypothesized that females would have a greater change in CVR across the intervention compared with males. Though sex differences in CVR have received minimal attention, the evidence available suggests the relationship between healthier cerebrovascular indices and higher aerobic fitness level are more apparent in females but not males. For example, a negative relationship between cerebral pulsatility index and $\dot{V}O_{2}\max$ was observed in females after correcting for age, but not in males (Zeller et al., 2022). In addition, the degree of middle cerebral artery blood velocity decline, and increase in pulsatility index observed with aging is greater in females (Alwatban et al., 2021). Given that cerebral blood velocity declines faster in females compared to males this may allow for greater improvement in cerebrovascular indices in females.

Recently, females were shown to have greater reductions in all‐cause mortality than men for the same amount of self‐reported leisure‐time physical activity (Ji et al., 2024). This increased sensitivity to the beneficial effects of physical activity may be in part due to females having more Type I oxidative muscle fibers than men, which may allow females to have a greater capacity for endurance training and recovery (Haizlip et al., 2015). Taken together, the evidence available to date led us to hypothesize that females will have greater changes in perfusion across the intervention compared with males.

Given that aerobic exercise improves vascular regulation (Guadagni et al., 2020), we hypothesized that a 6‐month aerobic exercise intervention would increase CBF and CVR measures in older adults. Further, we hypothesized that changes in perfusion and CVR in these structures would be positively correlated with changes in cognitive test scores.

## 2 METHODS

### 2.1 Ethical approval

These data were collected as part of the BIM study (Guadagni et al., 2020; Tyndall et al., 2013) which was approved by the Conjoint Health Research Ethics Board (REB14‐2284) at the University of Calgary. Participants were provided an overview of the experimental protocol and gave their written informed consent. The study was conducted in accordance with the Declaration of Helsinki, with the exception that it was not registered in a publicly accessible database before participant recruitment.

### 2.2 Participants

This study's participants were recruited from the BIM cohort, a quasi‐experimental study that evaluated cardiovascular and cerebrovascular physiology before and after a 6‐month aerobic exercise intervention (Guadagni et al., 2020).

Inclusion criteria included a body mass index (BMI) of <35 kg/m^2^, ability to independently walk up and down 20 stairs and no history of cardiovascular disease (CVD), type I diabetes mellitus, or respiratory, neurological or cognitive diseases. Participants also needed to be non‐smokers for the last 12 months, free of major surgery or trauma in the past 6 months, and to have had written permission from their primary health care professional to take part (Tyndall et al., 2013). No history of CVD was specified as no prior self‐reported history of myocardial infarction, chronic heart failure, stroke, or transient ischemic attack. Participants were excluded if they had angina, arrhythmia, or valve disease. In addition, prospective participants had to meet the following self‐reported sedentary criteria: to have not engaged in >30 min of moderate exercise, more than 4 days per week, and to have limited their intense exercise to no more than 20 min, 2 days per week. These criteria were determined in a telephone interview. Those who met these inclusion criteria performed an on‐site screening visit which included a cognitive assessment on which a score of ≥24 on the Montreal Cognitive Assessment (MoCA) was required. Self‐reported medical history and medications were recorded.

Participants included in this study are a convenience sample of participants who participated in a sub‐study of Brain in Motion that included undergoing two brain MRIs (*n* = 27). Each participant underwent one MRI session before the exercise intervention and another after the exercise intervention. Subjects included in the present study were those who participated in the exercise intervention and completed the optional MRI. Biological sex is defined by physiological and biological traits associated with reproductive anatomy and hormonal function. Participants were dichotomized as male or female with reference to their biological sex assigned at birth. All female participants in this sub‐study were post‐menopausal.

### 2.3 Exercise intervention

After enrolment into the study, there was a six‐month control period. Participants had a cognitive function assessment at month 0 (entry point into the study) and month 6 (at the end of the control period, immediately prior to the 6‐month aerobic exercise intervention). The intervention was a 6‐month aerobic exercise training of walking/running consisting of a 5‐min warm up, then aerobic fitness increasing from 20 min to 40 min per session, with relative intensity increasing to 60%–70% of participants' maximal heart rate reserve at least 3 times per week (Tyndall et al., 2013). The aerobic exercise was followed by 5 min of cool down and stretching. Compliance to the intervention was defined as attending more than 85% of the in‐person sessions, which has been demonstrated to be an optimal attendance percentage for observing cognitive improvements (Hall et al., 2019; Sáez de Asteasu et al., 2017). When a participant was unable to attend a supervised training session, they were asked to make up the missed session by performing aerobic exercise in their own time and to record these unsupervised make up sessions, along with any additional exercise they performed in their own time, in a workout diary. The average time from the last exercise session to the post‐intervention MRI visit was 28 days (range = 37 days before the end of the intervention to 116 days after the intervention, median = 34 days, interquartile range = 43 days).

Participants were asked to continue exercising until all post‐intervention assessments were completed. Their $\dot{V}O_{2}\text{peak}$ and MRI assessments were scheduled as close as possible to one another to ensure that measurements were accurate and meaningful. The median number of days between MRI and $\dot{V}O_{2}\text{peak}$ assessments were 20 days.

### 2.4 fMRI hypercapnia CVR protocol

BOLD fMRI was used to measure CVR. Prior to starting the CVR BOLD acquisition, the resting end‐tidal partial pressure of CO_2_ (Petco_2_) was determined while the participant breathed room air. Next, BOLD acquisition started with a 5‐min air‐breathing baseline, followed by 2 min of hypercapnia and a final minute of recovery. For the hypercapnic challenge, participants were instrumented with an oxygen diffuser mask through which 100% CO_2_ was administered to the participant at a flow rate sufficient to increase PETCO_2_ to +8 mmHg above the participant's air‐breathing baseline values. A nasal cannula was used to continuously sample respiratory gases for analysis by a dual O_2_‐CO_2_ analyzer (Normocap, Datex Ohmeda, Louisville, KY). Participants were instructed to exhale via their nose.

### 2.5 MRI acquisition

Data were collected using a 3 Tesla General Electric Discovery 750 with a 12‐channel head coil. A structural 3D IR‐FSPGR T_1_‐weighted image was acquired using the following parameters: TR = 6.7 ms, TE = 2.9 ms, flip angle = 10°, TI = 650 ms, field of view = 256 × 256 mm, voxel size = 1 mm^3^, receive bandwidth = 244 Hz/pixel.

Functional MRI was performed using a sequence sensitized to BOLD contrast with T_2_*‐weighted Gradient Echo‐Echo Planar Imaging (GRE‐EPI), TR = 2286 ms, TE = 30 ms, flip angle = 80°, 64 × 64 matrix, ASSET acceleration factor = 2, 43 oblique slices aligned to the anterior–posterior commissure plane (interleaved, bottom‐up slice order), 3.5 mm^3^ voxel size, 7813 Hz/pixel receive bandwidth.

Resting CBF was quantified using pseudo‐continuous arterial spin labelling (pCASL) with a 3D Fast Spin Echo (FSE), stack‐of‐spirals, background‐suppression on, and the following parameters: TR = 4745 ms, TE = 10 ms, 240 × 240 mm FOV, eight spiral arms with 512 points per arm, effective in‐plane resolution 3.64 × 3.64 mm^2^, 30 5‐mm slices, 977 Hz/pixel receive bandwidth, no acceleration, label duration 1500 ms, post‐label delay 2025 ms, 2 averages. The labelling location was 20 mm below the first inferior slice within the field‐of‐view.

### 2.6 MRI processing

Preprocessing of MR images was performed using the FMRIB's Software Library (FSL, University of Oxford, Oxford, UK) version 6.0.3. The following analyses were completed with the FMRI Expert Analysis Tool (FEAT) and a brain background threshold of 10%. Motion correction was applied using FSL's Motion Correction using FMRIB Linear Image Registration Tool (MCFLIRT). Slice timing correction was applied to the images. Brain extraction was applied to the functional images, using the brain extraction tool (BET). Spatial smoothing was performed using a full‐width at half max of 5 mm. A boxcar representation was used to model the timing of the hypercapnic challenge. It was convolved with a gamma function (standard deviation = 30 s, lag = 30 s) to resemble a hypercapnia hemodynamic response curve. Temporal derivatives (covariates to account for time to peak signal) were applied to account for slight variations in the onset of the stimulus. A linear nuisance regressor was added as an additional confound variable to model linear drift. FILM pre‐whitening was selected. Voxels with BOLD signal changes greater than 10% were removed to ensure that the BOLD signal change was originating from functional tissue and not large draining veins (Menon, 2002). Cerebrovascular reactivity was calculated as follows: (Xu et al., 2023):

$$\begin{array}{c} \text{Cerebrovascular reactivity}=\\ \left(\frac{\left(\frac{\text{Contrast of parameters estimate for baseline signal}\;\text{to hypercapnia}}{\text{baseline}}\right)\times 100}{\left({\text{PetCO}}_{2}\;\mathrm{at}+8\;\left(\text{mmHg}\right)\right)-\left({\text{PetCO}}_{2}\;\mathrm{at}\;\text{baseline}\;\left(\text{mmHg}\right)\right)}\right) \end{array}$$

[PETCO_2_: end‐tidal partial pressure of carbon dioxide].

Breathing frequency and tidal volume were not controlled but were monitored continuously via respiratory gas tracing (LabChart software, ADInstruments) and a pressure transducer to which the respiratory gas sampling line was connected. The end‐tidal values were identified as the local maxima of the CO_2_ waveforms. Baseline PETCO_2_ was calculated as the average of the last 30‐s of the 5‐min air breathing baseline period while PETCO_2_ during hypercapnia was calculated as the average of the final 30‐s of the hypercapnic period. This corresponded to 6–7 breaths in each 30‐s period. Full details of the gas delivery system and respiratory circuit are described in references (MacDonald et al., 2018; Tancredi et al., 2014).

T_1_‐ weighted images were brain extracted outside of FEAT using BET. Each structural image was registered to the MNI152 2 mm brain template using nonlinear registration with a warp resolution of 10 mm. To restrict analyses to gray matter, a gray matter mask was created on a per‐subject basis. The participant's skull stripped structural data were segmented into gray matter, white matter, and cerebrospinal fluid (CSF), using FMRIB's Automated Segmentation Tool (FAST). The gray matter masks were corrected for white matter hyperintensity volumes that were misclassified as gray matter. This was done using the FSL package Brain Intensity AbNormality Classification Algorithm (BIANCA) (Griffanti et al., 2016). The masks were co‐registered to the functional data using Statistical Parametric Mapping (SPM, version 12) in MATLAB. A threshold of <50% partial volume estimate (PVE) of gray matter or white matter was applied to the masks before binarization. CVR masks were multiplied by the gray masks, and CVR in the gray matter was reported as the average for all nonzero voxels. Gray matter volume was reported as the mean voxel PVE of the gray matter PVE map multiplied by the volume of all nonzero voxels in T1 space.

For ASL images, CBF maps in mL/100 g/min automatically generated by the scanner were used. The gray matter masks were co‐registered to the CBF maps using SPM12. The masks were multiplied by the CBF maps, and the average of all nonzero voxels was reported.

For regions‐of‐interest analyses, masks of the left pars triangularis, insula, and hippocampus were made using the Juelich Histological Atlas or the Harvard‐Oxford cortical structure atlas. The regions were isolated by applying a binarized version of the mask to the CBF data of each participant, pre and post exercise.

### 2.7 Cardiorespiratory fitness test

Maximal aerobic capacity ($\dot{V}O_{2}\text{peak}$) was assessed on a motorized treadmill using the modified Bruce protocol (Bruce et al., 1973; McInnis & Balady, 1994) once before the exercise intervention (pre), and once after 6 months of the intervention (post). Briefly, for each test workload was increased until volitional exhaustion as determined by the participant's indication of inability to continue at the current work rate. Criteria for determining $\dot{V}O_{2}\text{peak}$ were based on guidelines from the American College of Sports Medicine (ACSM) and included the appearance of a plateau in $\dot{V}O_{2}\text{peak}$ of <2 mL/kg/min, a respiratory exchange ratio ≥1.15, and an age predicted maximal heart rate of at least 210 – (age × 0.65).

The sedentary model was used to calculate predicted $\dot{V}O_{2}\text{peak}$ for females (predicted sedentary $\dot{V}O_{2}\text{peak}$ = 43.82–(0.35 × age)) (Fitzgerald et al., 1997). Predicted $\dot{V}O_{2}\text{peak}$ using the sedentary model for males was calculated (predicted sedentary $\dot{V}O_{2}\text{peak}$ for males = 54.2–(0.40 × age)) (Wilson & Tanaka, 2000). Actual $\dot{V}O_{2}\text{peak}$ was divided by the predicted $\dot{V}O_{2}\text{peak}$ and multiplied by 100 to obtain a percent of predicted value.

### 2.8 Blood pressure and capillary blood sample

Arterial blood pressure was measured using a beat‐by‐beat finger pulse photoplethysmography (Finometer, Finapres Medical Systems, Amsterdam, The Netherlands) (Guelen et al., 2008; Imholz et al., 1998). The systolic and diastolic blood pressures were monitored continuously. Values were corrected with a manual brachial blood pressure measurement. The values reported here are at rest, during room air breathing. Average diastolic blood pressure collected from the manual brachial measurement was divided by the average photoplethysmography derived blood pressure measurement, which produced a correction factor. The diastolic and systolic blood pressures from the photoplethysmography were multiplied by the correction factor (Beaudin et al., 2019). Systolic and diastolic blood pressure was averaged across 10‐min of a rest, room air breathing collection period. Mean arterial pressure was calculated as the diastolic blood pressure + 1/3 (systolic blood pressure – diastolic blood pressure). Heart rate was collected using a 3‐lead Electrocardiogram (Micromon 7142 B, Kontron Medical, Milton Keynes, UK). The signal was extracted as a 10‐volt signal from the R‐wave. These data were recorded and saved in real time as part of our data acquisition system. The occurrence of the R‐wave was recorded and time‐aligned with all other physiological data. The time of each heart beat was then exported to a file, and beat‐by‐beat data were averaged across a 10‐min rest period (Tyndall et al., 2016). Capillary blood samples were taken from the middle finger, and then analyzed for hematocrit using a radiometer (Radiometer ABL 800, Denmark) (Tyndall et al., 2013). Hematocrit values collected at month 0 were used for analysis.

### 2.9 Cognitive tests

A cognitive testing battery was administered to participants at study entry (month 0), at a second baseline that was immediately prior to the exercise intervention (month 6), and after the exercise intervention (month 12). The verbal fluency portion of the Delis‐Kaplan Executive Functioning System (D‐KEFS) and the scores from the Buschke Selective Reminding Test were used for analysis in this study. Two separate baselines across the control period were used to test for any practice effects. The scores from the second baseline immediately prior to the intervention were used as the measures of interest for the present study.

The measure of interest for analyses in the present study was the scores for letter fluency, categorical fluency, and categorical switching. In the Buschke Selective Reminding Test, participants were asked to recall a list of 12 words that were read to them, immediately following each reading (immediate recall) and 30 min later (delayed recall). The delayed recall score was used in the analysis to investigate episodic verbal memory (retrieval of consolidated verbal information). Age corrected verbal fluency scores were used in the analysis. The delayed recall scores were age and sex corrected and converted to *z*‐scores for analysis.

### 2.10 Statistical analysis

Student's paired samples *t*‐tests were used to assess if there were any changes in cerebrovascular measures, and $\dot{V}O_{2}\text{peak}$ from pre‐ to post‐intervention. In addition to $\dot{V}O_{2}\text{peak}$ normalized by body weight, we report a percent predicted $\dot{V}O_{2}\text{peak}$ value which normalizes the data set and makes it possible to compare different age and sex groups on the same scale (Stelken et al., 1996). Mixed factorial two‐way, repeated measures, analyses of covariance (ANCOVA) were implemented to assess the effect of aerobic exercise and sex on left pars triangularis, insula, and hippocampal CBF, and CVR, while controlling for total intracranial volume (ICV), mean arterial blood pressure, and baseline gray matter cerebral blood flow. We also tested a model that controlled for the participant's compliance to the intervention, a model that included the time between their last exercise session to their MRI collection date, a model that included antihypertensive medications as a covariate, a model that included hormone replacement therapy usage, and a model that included body mass index (BMI) as a covariate. Since the BOLD signal relies on the quantity of T_2_* signal and is dependent on the volume of red blood cells in the blood, hematocrit variability between participants at baseline, or hematocrit changes from pre‐ to post‐ intervention, would confound BOLD signal changes (Xu et al., 2018). We therefore also tested a model which controlled for participant hematocrit at baseline (month 0) when investigating BOLD CVR in the brain regions. Separate ANCOVAs were run for the dependent variables CBF and CVR, and for each of the brain regions. For each ANCOVA, the repeated measures factor was time (pre and post intervention), and the between subject's variable was sex (male of female). After the first 12 scans at pre‐intervention, there was an MRI scanner upgrade, we therefore also controlled for pre‐ or post‐ upgrade status. Partial eta squared ($\eta_{p}^{2}$) was reported for the effect size for the ANOVA, and Cohen's *d* was used to report pairwise comparison effect size. Pairwise comparisons were evaluated at a Bonferroni corrected alpha level of *p* = 0.0125. The CBF and CVR values for each brain region met the assumption of normality (*p* > 0.05, assessed using the Shapiro–Wilk test). To assess if the change in verbal fluency and delayed recall scores from the second baseline (month 6) to post‐intervention were correlated with change in CBF and CVR in the brain regions, partial correlations were completed, controlling for age, years of education, and mean arterial blood pressure. Results for continuous and normally distributed data are presented as means ± standard deviation. Data analysis was completed in IBM SPSS for Mac version 26.0.

## 3 RESULTS

### 3.1 Participant characteristics

A total of 206 individuals completed the exercise program in the BIM study (Guadagni et al., 2020), of which 32 completed a pre‐intervention MRI. Five participants subsequently withdrew, with the remaining 27 completing both a pre‐ and post‐aerobic exercise MRI (age range 57.5–85.3 years pre‐intervention, 12 females; Table 1). Six participants were taking antihypertensive medication during the study. Two female participants reported they were on hormone replacement therapy (estradiol, one on vaginal and the other on oral administration).

**TABLE 1:** Demographic and health metrics, paired samples *t*‐test results.

	CVR sample (*n* = 26, females = 11)					CBF sample (*n* = 27, females = 12)				
	Pre intervention phase	Post intervention phase	*t*	Cohen's *d*	*p* Value	Pre intervention phase	Post intervention phase	*t*	Cohen's *d*	*p* Value
Age (years)	66.5 ± 7.2	67.2 ± 7.2	−34.73	0.09	1.11E‐22	67.1 ± 7.6	67.8 ± 7.7	−35.53	0.09	1.44E‐23
Body mass index (BMI) (kg/m^2^)	25.8 ± 3.2	25.5 ± 3.2	3.15	0.03	0.004	25.7 ± 3.2	25.4 ± 3.2	3.34	0.09	0.003
Systolic pressure at baseline (mmHg)	124.7 ± 15.8	123.5 ± 16.7	0.43	0.07	0.672	126.1 ± 17.3	125.1 ± 18.3	0.39	0.06	0.700
Diastolic pressure at baseline (mmHg)	75.9 ± 8.7	73.5 ± 10.0	1.28	0.26	0.212	75.9 ± 8.6	73.7 ± 9.8	1.20	0.24	0.242
Mean arterial pressure (mmHg)	92.2 ± 9.7	90.2 ± 11.0	1.072	0.19	0.294	92.6 ± 9.8	90.8 ± 11.3	0.17	0.17	0.328
In person track sessions attended (range, mean)	22–78, 54.2					22–78, 54.0				
Make‐up logbook sessions (range, mean)	0–39, 10.2					0–39, 10.2				
Total number of sessions completed (range, mean)	22–105, 64.4					22–105, 64.2				
Years of education completed	16.2 ± 2.5					16.2 ± 2.5				

*Note*: Values are mean ± standard deviation unless otherwise stated.

Demographics of this convenience sample did not differ from the larger BIM study, except for the current study's participants having a lower BMI (25.7 ± 3.2 versus 27.2 ± 3.7, *p* = 0.046; Table S1). Eight out of the 27 participants in this sample did not attend more than 85% of the exercise sessions and were deemed noncompliant. Age, sex, and attendance of these participants are shown in Table S2.

Due to poor BOLD image quality, one participant was excluded from CVR analysis, resulting in a final sample size of 26 participants. One participant did not complete the $\dot{V}O_{2}\text{peak}$ test at pre‐ intervention due to atrial fibrillation during the test. Therefore, $\dot{V}O_{2}\text{peak}$ analyses included 25 participants. Hematocrit values were collected for 25 out of the 26 participants in the BOLD CVR analysis. The temporal signal to noise ratio of the BOLD data was not different between those whose data spanned the upgrade and those who did not.

Cognitive test scores at month 0 (study enrolment) and month 6 (immediately prior to the start of the aerobic exercise intervention) were not different (*p* > 0.05 for all tests), indicating no practice effects.

At study enrolment, there were no differences between males and females in age (*p* = 0.122) or relative $\dot{V}O_{2}\text{peak}$ in ml/kg/min (*p* = 0.570). However, pre‐intervention pp. $\dot{V}O_{2}\text{peak}$ was higher in females (*p* = 0.023; Table S3). There were no differences in resting heart rate (*p* = 0.841; Table S3) or systolic blood pressure between females and males (*p* = 0.359; Table S3). Females had a significantly lower diastolic blood pressure (*p* = 0.01) at rest compared to males before the exercise intervention. After the exercise intervention, diastolic blood pressure was not different between the sexes (*p* = 0.174; Table S3). The mean exercise duration per week was not different between males and females (*p* = 0.850; Table S3).

### 3.2 Fitness level, CVR, and CBF

**FIGURE 1:**
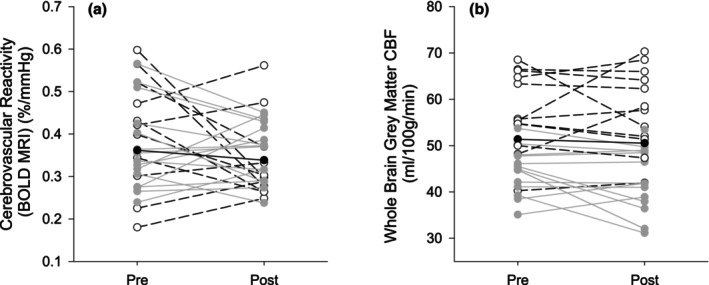
Changes in cerebrovascular indices across the intervention shown as regression lines for (a) cerebrovascular reactivity measured using BOLD, *n* = 26 (b) whole brain gray matter cerebral blood flow measured using ASL, *n* = 27. Females are depicted as open circles and black dashed lines, and males are depicted as gray circles and gray lines. The mean is represented by the black solid circle and solid black line.

Maximal aerobic capacity significantly improved by 6.7% from pre‐intervention to post‐intervention (Table 2). None of the cerebrovascular measures changed significantly from pre‐ to post‐exercise intervention (Table 2 and Figure 1).

**TABLE 2:** Cerebrovascular measures pre and post intervention.

Test	Pre		Post		Paired *t* test	Cohen's *d*	*p* Value	Mean difference [95% confidence interval]
	Sample size	Mean ± standard deviation	Sample size	Mean ± standard deviation				
BOLD MRI (for CVR) (% Signal Change/PETCO_2_)	*n* = 26	0.38 ± 0.11	*n* = 26	0.35 ± 0.08	1.48	0.32	0.151	0.03 [−0.13 to 0.08]
ASL MRI (for gray matter resting CBF) (mL/100 g/min)	*n* = 27	51.1 ± 9.6	*n* = 27	49.5 ± 10.7	1.26	0.16	0.217	1.56 [−0.97 to 4.08]
$\dot{V}O_{2}\text{peak}$ (ml/kg/min)	*n* = 26	26.5 ± 5.5	*n* = 26	28.5 ± 6.1	−5.01	−0.34	3.61E‐05	1.99 [−2.80 to 1.17]
Percent predicted $\dot{V}O_{2}\text{peak}$ assuming sedentary	*n* = 26	110.4 ± 19.5	*n* = 26	119.4 ± 18.9	−5.78	−0.47	5.06E‐06	9.03 [−12.25 to 5.81]
PETCO_2_ MRI baseline (mmHg)	*n* = 26	32.8 ± 3.2	*n* = 26	32.5 ± 3.6	0.58	0.09	0.570	0.24 [−0.61 to 1.07]
PETCO_2_ MRI change (mmHg)	*n* = 26	6.2 ± 2.2	*n* = 26	7.3 ± 2.5	−2.03	−0.45	0.053	1.04 [−2.11 to 0.01]

*Note*: Values are mean ± standard deviation.

### 3.3 Brain region a priori sex differences

Whole brain gray matter volume fraction was not different between males and females pre‐intervention (after controlling for ICV and blood pressure and scanner upgrade females gray matter volume = 586.97 ± 15.75 mL, males gray matter volume 582.20 ± 15.488 mL; *F*[1,22] = 0.54, *p* = 0.470, $\eta_{p}^{2}$ = 0.02, 95% CI [−8.70 to 18.25]).

**FIGURE 2:**
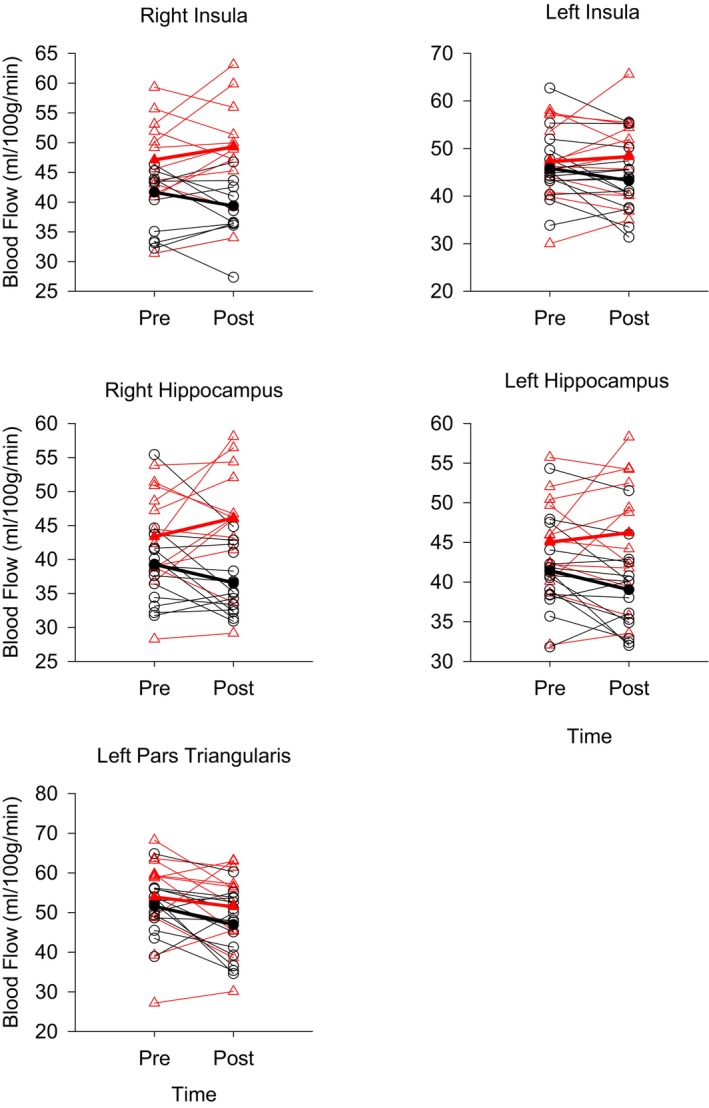
Raw, uncorrected region of interest blood flow shown by sex, pre and post exercise intervention, shown as regression lines, *n* = 27. Red open triangles and dashed line indicate female values, and open black circles and solid black line indicate male values.

Differences in CBF and CVR between sexes and changes with the aerobic exercise intervention are reported in Table 3. There was no effect of the intervention on whole brain resting CBF in the gray matter, or any brain region, except for the left pars triangularis. Furthermore, there was no effect of sex in CBF within any brain region (Table 3). Although, after controlling for ICV, mean arterial blood pressure, and scanner upgrade, whole brain resting CBF in gray matter was higher in females compared to males at both pre‐exercise and at post‐exercise intervention (Table 3). Intervention and sex interactions were observed for all brain regions but not for whole brain cerebral blood flow (Table 3 (corrected statistics), and Figure 2 (raw perfusion values)). These results did not change when using separate models that controlled antihypertensive medication use, or days in between their last exercise session to their MRI appointment. When including compliance to the intervention, the interaction effect in the left insula (*p* = 0.096), and left hippocampus was no longer observed (*p* = 0.065). Similarly, when using hormone replacement therapy as a covariate, the interaction effect was no longer observed in the left insula (*p* = 0.065) and left hippocampus (*p* = 0.079). When including BMI as a covariate in the model, the sex and intervention interaction was no longer observed for the left hippocampal region (*p* = 0.088), and the left insula (*p* = 0.081). Pairwise comparisons revealed that females remained the same in their resting CBF, while males declined in CBF in the right hippocampus, and left pars triangularis from pre to post intervention (Table 3). There was no effect of the intervention, sex, and no interaction on whole brain gray matter BOLD CVR, or CVR within brain regions (Table 3). These results were similar in models that controlled for compliance to the intervention, anti‐hypertensive medication use, days in between their last exercise session to their MRI appointment, BMI, hormone replacement therapy, or hematocrit.

**TABLE 3:** Results for the repeated measures ANOVAs performed for each region of interest, cerebral blood flow (CBF), and cerebrovascular reactivity (CVR).

Region of interest	Fixed factor	CVR (*n* = 26)			CBF (mL/100g/min) (*n* = 27)			Post hoc comparisons: Pre‐intervention CBF						Post hoc comparisons: Post‐intervention CBF					Post hoc comparisons: Within sex CBF				
								Between sex pre‐intervention^b^						Between sex post‐intervention^b^					Within sex pre to post intervention				
		*F*	*p*	$\eta_{p}^{2}$	*F*	*p*	$\eta_{p}^{2}$	Sex	Mean ± SD	Mean difference [95% CI]	*t*	Cohen's d	*p*	Mean ± SD	Mean difference [95% CI]	*t*	Cohen's d	*p*	Sex	Mean difference [95% CI]	*t*	Cohen's d	*p*
Gray matter	Sex	0.00	0.937	0.00	16.21	6.61E‐04^a^	0.45	F	58.2 ± 9.3	12.7 [4.2 to 21.2]	3.11	1.58	0.006^a^	59.5 ± 9.6	18.0 [9.2 to 26.8]	4.26	1.90	3.93E‐04^a^	F	1.4 [−3.2 to 6.0]	0.62	−0.15	0.540
								M	45.5 ± 9.3					41.6 ± 9.3					M	−3.9 [−8.0 to 0.08]	−2.04	0.42	0.054
	Intervention	1.21	0.286	0.06	0.24	0.629	0.01																
	Interaction	0.32	0.563	0.03	2.48	0.131	0.11																
Right insula	Sex	0.01	0.912	0.00	0.80	0.381	0.04	F	42.7 ± 7.1	2.5 [−9.36 to 4.35]	0.76	0.37	0.453	48.1 ± 8.0	7.9 [0.2 to 15.6]	2.14	1.01	0.045	F	5.4 [0.8 to 10.0]	2.45	−0.71	0.024
								M	45.2 ± 6.3					40.3 ± 7.4					M	−4.9 [−8.9 to −1.0]	−2.62	0.71	0.017
	Intervention	0.87	0.364	0.05	1.00	0.328	0.05																
	Interaction	0.75	0.399	0.04	8.65	0.008^a^	0.31																
Left insula	Sex	0.07	0.799	0.00	0.04	0.843	0.00	F	44.1 ± 6.8	4.2 [−2.3 to 10.8]	1.34	0.64	0.195	47.0 ± 10.6	2.8 [−7.6 to 12.9]	0.55	0.26	0.590	F	2.8 [−1.1 to 6.8]	1.52	−0.33	0.145
								M	48.4 ± 6.5					44.3 ± 10.0					M	−4.3 [−7.4 to 0.7]	−2.52	0.47	0.021
	Intervention	1.08	0.313	0.06	0.43	0.522	0.02																
	Interaction	0.69	0.417	0.04	5.26	0.033^a^	0.22																
Right hippocampus	Sex	0.22	0.647	0.01	0.59	0.454	0.03	F	38.8 ± 4.2	4.2 [0.3 to 8.3]	2.10	0.99	0.048	44.8 ± 7.2	7.3 [0.35 to 14.3]	2.20	1.04	0.040	F	6.0 [1.4 to 10.7]	2.71	−1.01	0.014
								M	42.9 ± 4.1					37.5 ± 6.9					M	−5.4 [−9.4 to −1.5]	−2.86	0.95	0.010^a^
	Intervention	0.96	0.340	0.05	1.01	0.327	0.05																
	Interaction	0.14	0.716	0.00	10.40	0.004^a^	0.35																
Left hippocampus	Sex	0.17	0.686	0.00	0.80	0.383	0.04	F	42.5 ± 4.4	0.7 [−3.5 to 4.8]	0.23	0.45	0.658	45.2 ± 7.7	5.8 [−1.2 to 12. 8]	1.53	0.72	0.141	F	2.7 [−1.0 to 6.5]	1.49	−0.43	0.151
								M	43.5 ± 4.3					39.8 ± 7.3					M	−3.6 [−6.8 to −0.4]	−2.37	0.62	0.029
	Intervention	0.24	0.628	0.01	0.26	0.616	0.01																
	Interaction	0.04	0.850	0.00	4.84	0.040^a^	0.20																
Left pars triangularis	Sex	0.27	0.613	0.02	0.01	0.906	0.00	F	48.5 ± 7.4	8.2 [1.3 to 15.1]	2.18	1.03	0.040	53.5 ± 8.6	8.8 [1.3 to 16.4]	2.06	0.98	0.053	F	5.7 [1.6 to 9.7]	2.22	−0.62	0.039
								M	55.9 ± 7.0					45.3 ± 8.2					M	−11.4 [−14.8 to −7.9]	−5.56	1.39	2.29E‐05^a^
	Intervention	0.68	0.421	0.04	10.19	0.005^a^	0.35																
	Interaction	0.00	0.969	0.00	18.94	3.43E‐04^a^	0.50																

*Note*: All ANOVAs controlled for mean arterial blood pressure, baseline cerebral blood flow, scanner upgrade, and intracranial volume.

Abbreviation: CI, confidence interval.

^a^ Indicates significance at *p* < 0.05 for main effects, and *p* < 0.0125 for post hoc comparisons.

^b^ Between‐sex only post hoc comparisons performed.

### 3.4 Cognitive test and region of interest perfusion

The only cognitive test score that changed from pre‐ to post‐ intervention was the delayed recall trial from the Bushke Selective Reminding Test (Table S4). Overall, participants improved their performance on this test following the aerobic exercise intervention. Previously, the composite scores of the cognitive test scores across the intervention were reported for the larger BIM Study (Guadagni et al., 2020).

Partial correlations showed that CBF change in the right insula, right hippocampus, and left pars triangularis perfusion across the intervention was not associated with change in any of the cognitive test scores while controlling for age, years of education, and mean arterial blood pressure. Change in left insula and left hippocampal perfusion was negatively correlated with letter fluency (Table S5). Change in CVR within brain regions was also not correlated with change in cognitive test scores (Table S6).

## 4 DISCUSSION

This study investigated resting whole brain CBF and CVR in older adults across a 6‐month aerobic exercise intervention. While there was no main effect of sex on CBF within any brain regions, there was a sex‐by‐intervention interaction observed for all brain regions, which suggests that CBF responds to aerobic exercise training differently between females and males. Our second hypothesis was not supported as CBF and CVR remained constant across the intervention. CVR in the left pars triangularis and CBF in the left insula, post intervention, were negatively correlated with the delayed recall and letter fluency, respectively, which did not support our hypothesis.

### 4.1 Brain region sex differences with exercise

After controlling for BMI, the sex and intervention interaction was no longer observed in the left hippocampus and left insula. Greater BMI has been shown to be negatively correlated with cerebral blood flow (Amen et al., 2020; Willeumier et al., 2011). Males in our study had a greater BMI compared with females at pre and post intervention (Table S3). There was a significant sex and intervention interaction without correcting for BMI for left hippocampal perfusion (*p* = 0.040) and left insula perfusion (*p* = 0.033), but this interaction was not significant after adding BMI as a covariate (left hippocampus *p* = 0.162, left insula *p* = 0.081). BMI declined in males across the intervention (*p* = 0.008) but did not for females (*p* = 0.157). These results suggest that the reduction in BMI may have contributed to a reduced decline in left hippocampal and left insula perfusion across the intervention in males. A reduction in weight with aerobic exercise training reduces inflammatory biomarker levels, specifically the reduced expression of cytokines (monocytes CD14+CD16+ and TNFα production), and intracellular reactive oxygen species has been observed with exercise (Beavers et al., 2010). Furthermore, a strong contributor to chronic inflammation is TREM‐1, which has been found to be elevated in the blood of those with obesity (Subramanian et al., 2017). Greater BMI is related to chronic systemic inflammation, and this may reduce perfusion through TREM‐2‐MAPK signaling (Zhang et al., 2020).

Similar to whole brain sex differences observed in adults <70 years of age (Alisch et al., 2021; Juttukonda et al., 2021), female participants had higher resting CBF measured by ASL compared to males. In a recent study investigating sex differences in resting perfusion in young adults using ASL, females had consistently greater CBF compared to males across the triangular part of the inferior frontal gyrus (~20% greater in females), the thalamus (~36% greater in females), and the hippocampus (~31% greater) (Muer et al., 2024). Females also had greater perfusion in 67/68 brain regions investigated. Female participants in our study demonstrated a tendency to show an increase (right insula *p* = 0.024, right hippocampus *p* = 0.014, left pars triangularis *p* = 0.039) in perfusion in the same regions. We did not see a main effect of sex in the regions of interest with females having greater perfusion, which is consistent with findings of sex differences in perfusion disappearing after the age of 65 (Aanerud et al., 2017). Interestingly, cerebral metabolic rate of oxygen consumption is not different between the sexes (Aanerud et al., 2017). However, recent findings show that sex differences in metabolism exist in the retrosplenial cortex. Hypometabolism in this region predicts conversion from MCI to Alzheimer Disease (Terstege et al., 2025). Female mice have reduced metabolism in the retrosplenial cortex, which is not observed in male mice (Terstege et al., 2025). Female mice and female patient post‐mortem tissue showed less parvalbumin interneurons compared to male tissue (Terstege et al., 2025). Therefore, sex differences in response to interventions that would improve vascular function may be due, at least in part, to underlying sex differences in neuronal composition changes with aging.

There was no demonstrable effect of the intervention on CVR within any brain regions. These findings are similar to DuBose and colleagues' (2022), who found that there was no change in the CVR response (quantified using a breath hold challenge) measured using BOLD MRI across a 12‐week intervention of cycling at a moderate‐vigorous intensity in older adults (DuBose et al., 2022). In addition, we found no effect of sex on CVR in any of the brain regions while controlling for baseline CBF. In relation to cardiorespiratory fitness level, CVR changes have been found to be variable, with sex accounting for 7.08% of the heterogeneity, and mean arterial blood pressure explaining 36.8% (Smith et al., 2021). Differences between the sexes in cerebrovascular responses have been demonstrated and there is evidence that females have a greater response to blood flow increasing agents such as L‐arginine, compared with males in both the posterior cerebral artery and middle cerebral artery (Perko et al., 2011). Future investigations are needed to determine sex differences in CVR function across an aerobic exercise intervention.

### 4.2 Cognitive changes with aerobic exercise

We found that delayed memory performance on a selective reminding task was better post‐aerobic exercise with a small to medium effect size, while verbal fluency performance (letter fluency, categorical fluency, and categorical switching) did not change. Our results suggest that 6 months of aerobic exercise can improve memory independent of changes in CBF. These results are consistent with longitudinal exercise intervention studies, demonstrating that exercise interventions have the potential to improve memory in community dwelling older adults, with a small, but clinically meaningful effect on the change in executive functioning (Hoffmann et al., 2021).

While changes in perfusion do not necessarily affect improvements in cognitive function acutely (Smith & Ainslie, 2017), we hypothesized that chronic exercise would induce benefits to brain regions in older adults, possibly from brain vascularization, or from exerkine release thus modifying metabolic and inflammatory environments (Chow et al., 2022), reflected with cerebrovascular reactivity as a measure of vascular brain health. Both exercise and hypercapnia lead to an increase in nitric oxide (NO) production. A low pH causes chemoreflexes to upregulate the main synthesizer of cerebral NO, the gene encoding for the endothelial NO synthase (eNOS) protein. The production of NO activates cyclic guanosine monophosphate (cGMP) signaling pathways and vasodilation of neighboring smooth muscle cells (Lundberg & Weitzberg, 2022). eNOS is also affected by fitness status (da Silva et al., 2018). Availability of NO leads to improved arterial functionality (Tousoulis et al., 2012). Beneficial vascular modifications from exercise, at the level of the artery, can be attributed to the change in gene expression of key players in vasodilatory response such as eNOS, namely C‐type natriuretic peptide (CNP), and PGE‐EP2R/4R (Maeda et al., 2005).

### 4.3 Cerebrovascular changes with aerobic exercise

CBF and CVR remained constant across the aerobic exercise intervention. There is a possibility that the effect of aerobic exercise on CVR may depend on baseline fitness level, but in a non‐linear fashion. In a 3‐month aerobic exercise study in adults 50–79 years old, CVR assessed using BOLD imaging and a breath‐hold protocol was found to be non‐linearly related to $\dot{V}O_{2}\max$ (DuBose et al., 2022). CVR was positively associated with $\dot{V}O_{2}\max$ but only in areas of the default mode network in older adults with a $\dot{V}O_{2}\max$ less than ~28 mL/kg/min. The sample used in our study had an average $\dot{V}O_{2}\text{peak}$ of 26.5 mL/kg/min at pre‐, and 28.5 mL/kg/min post‐intervention, and we found no relationship between CVR with $\dot{V}O_{2}\text{peak}$ at pre‐ intervention. This could be attributable to the size of our cohort, as only the plateau region of the relationship DuBose and colleagues observed was drawn (DuBose et al., 2022).

Over the 6‐month aerobic exercise intervention, CBF and CVR remained constant while $\dot{V}O_{2}\text{peak}$ improved. While we hypothesized that CBF and CVR in each of the brain regions would increase across the exercise intervention, indicating a positive benefit of exercise to offset CBF declines with age, we did not observe any differences. Similarly, Burley and colleagues found no association between CVR and $\dot{V}O_{2}\max$ in their sample of 15 older adults using BOLD MRI (Burley et al., 2021).

CBF response to the intervention could also depend on baseline fitness level, with CBF negatively related to $\dot{V}O_{2}\max$ in highly fit younger individuals (Burley et al., 2021; Krishnamurthy et al., 2022). A possible theory for the negative relationship is that lower perfusion is indicative of more efficient neuronal activity, which may lower the threshold of ease of perfusion to meet metabolic demands (Krishnamurthy et al., 2022). This theory may also explain the negative relationships we found between change in letter fluency and perfusion in the left insula although the relationships we reported here were weak.

Although we did not observe increases in whole brain CBF or CVR after the intervention, we cannot conclude that the aerobic intervention is not effective in improving cerebrovascular health. We demonstrated that there are regional improvements in CBF across the intervention in females. The constant whole brain CBF observed in this sample may be interpreted as a stable CBF with aging instead of a decline; however, the lack of a nonexercise control group limits our ability to make this interpretation. The whole brain and regional CBF values reported in this study likely reflect a level of CBF that is within a range of being healthy and adequate for neuronal functioning, given that the older adults in the study are non‐cognitively impaired.

It is likely that changes in cerebrovascular function such as reduced perfusion may precede symptoms of cognitive decline (Wolters et al., 2017). Lower perfusion is associated with accelerated cognitive decline (Wolters et al., 2017), and improvements in cognition across an aerobic exercise intervention are explained, in part, by improvements in cerebrovascular health (Brown et al., 2010; Guadagni et al., 2020). Furthermore, sex differences in regional perfusion of these areas have not been reported, and females and males display differences in trajectories and severity of symptoms with Alzheimer's dementia progression (Arenaza‐Urquijo et al., 2024). If females and males respond differently in their cerebrovascular function to an aerobic exercise intervention, preventative therapies may need to be tailored to each sex to produce effective reductions in risk at a pre‐clinical stage.

### 4.4 Strengths and limitations

A limitation of this study is the small sample size, particularly for the investigation of sex differences in the brain regions. The significant sex and intervention interaction differences we found should be interpreted with caution. With the small sample size, there is a possibility that the sample is heterogeneous, and the effect size observed may be overestimated. The sample size also restricts our ability to generalize our findings, and although it is comparable to previous neuroimaging studies (Szucs & Ioannidis, 2020), further research is required to confirm that the sex differences across the aerobic exercise intervention are supported with a larger sample. Due to the small sample size, the analyses are susceptible to Type I and II statistical errors; therefore, our results need to be confirmed within a larger cohort.

Another limitation is that we did not have a non‐exercising control group. Nonetheless, the repeated measures study design permitted each participant to serve as their own control for the 6‐month period preceding the exercise intervention. Importantly, Spencer and colleagues found that the 6‐month period preceding the exercise intervention, $\dot{V}O_{2}\max$ and cerebrovascular responses to hypercapnia using transcranial Doppler ultrasound of the MCA remained stable in the Brain in Motion participants (Spencer et al., 2015).

Although the sample within the current study had similar demographics to the larger BIM cohort, we cannot rule out sampling bias as participants who were pro‐actively interested in participating in this BIM sub‐study may reflect a sample of healthy older adults who are highly motivated to lead healthy lifestyles, with less room for improvement (i.e., ceiling effect) compared to an at‐risk sample with vascular complications. This is reflected in the mean percent predicted $\dot{V}O_{2}\text{peak}$ for this cohort being greater than 100% (Table 2). In addition, the community dwelling sample was mostly highly educated (mean years ± SD = 16.5 ± 2.5) and mostly Caucasian, therefore limiting generalizability to more diverse, less educated populations. A small portion of the aerobic exercise sessions were made up on the participant's own time outside of the guided session with the exercise trainer, if the participant was unable to attend the session. This allows for the possibility of inaccuracy in the number of sessions attended given the subjective report.

We included the noncompliant participants in our analyses as this study consisted of a convenience sample of community dwelling older adults; in addition, we tested a model that controlled for the participants' compliance to the intervention. Compliance explained some of the heterogeneity of the results, as we no longer observed interaction effects for the left insula and left hippocampus when controlling for compliance. This may indicate that compliance to the intervention, in part, explains some of the interaction effects (sex differences in CBF across the intervention). Future studies should investigate the dosage of exercise necessary for changes in cerebral perfusion, as the individuals who were noncompliant reflect community dwelling older adults who may experience barriers to exercising consistently.

Finally, oxygen extraction fraction and cerebral metabolic rate of oxygen (CMRO_2_) contribute to the BOLD signal and may be a more direct measure of neurovascular function and cerebrovascular health (Moradi & Buxton, 2013). Therefore, future studies should investigate these indices in relation to cardiorespiratory fitness and aging.

The main strengths of the study were the use of MRI to measure cerebrovascular outcomes, the use of carbon dioxide as a stimulus to measure cerebrovascular reactivity, and $\dot{V}O_{2}\text{peak}$ to measure cardiorespiratory fitness in older adults. Our study addresses a critical gap in the effects of exercise on cerebrovascular health for older adults by investigating sex differences in hippocampal, insula and left pars triangularis cerebrovascular outcomes across an aerobic exercise intervention.

## 5 CONCLUSIONS

A 6‐month aerobic exercise intervention improved maximal aerobic capacity in older adults. Whole brain CBF and CVR remained constant across the intervention. There was an effect of the intervention on perfusion in the left pars triangularis. We provide preliminary evidence to suggest that females and males differ in their cerebral blood flow changes across an aerobic exercise intervention in the regions of the hippocampi, insula, and left pars triangularis.

## AUTHOR CONTRIBUTIONS

**Alison M. H. Donald:** Conceptualization; data curation; formal analysis; investigation; methodology; software. **Rebecca J. Williams:** Data curation; formal analysis; investigation; methodology; resources; software; supervision. **Andrew E. Beaudin:** Conceptualization; data curation; formal analysis; investigation; methodology; project administration; resources; software; supervision. **Erin L. Mazerolle:** Conceptualization; data curation; investigation. **Brandy L. Callahan:** Conceptualization; methodology; supervision. **G. Bruce Pike:** Conceptualization; data curation; funding acquisition; investigation; resources; supervision. **Marc J. Poulin:** Conceptualization; data curation; funding acquisition; investigation; project administration; resources; supervision.

## FUNDING INFORMATION

AD is supported by funding from Brenda Strafford Foundation Chair in Alzheimer Research (BSFCAR) (project number RT751959). MJP holds the BSFCAR. AD is also supported by the Alberta Innovates Graduate Studentship Data‐Enabled Innovation (Principal Investigator (PI): MJP), a Brain CREATE grant from the National Sciences and Engineering Research Council (NSERC) of Canada (PI: MJP) (project number 10025902), and a Canadian Institutes of Health Research (CIHR) Project grant. EM is supported by an Alberta Innovates Health Solution and NSERC Postdoctoral Fellowships. AEB is supported by Postdoctoral Fellowships from the Canadian Alzheimer Society, Campus Alberta Neuroscience, and the CIHR.

## CONFLICT OF INTEREST STATEMENT

The authors declare no potential conflicts of interest.

## Supporting information


Tables S1–S6.

## Data Availability

The Tables S1–S6 for this article will be made available online. Data can be made available on reasonable request.
