# Supplementary material for: Sex differences in cerebrovascular function across an aerobic exercise intervention in older adults using MRI: Results from the Brain in Motion study
Source: Physiol Rep. 2026 Apr 17;14(8):e70880. doi: 10.14814/phy2.70880 (PMC13090539; doi:10.14814/phy2.70880)
Supplement: Supplementary file 1 — Tables S1–S6. [file PHY2-14-e70880-s001.docx]

|  | *df* | MRI sample Mean ± SD | Larger Brain in Motion sample Mean ± SD | *t* | Cohen’s *d* | *p*-value | | Mean Difference | | *95% CI* |
| --- | --- | --- | --- | --- | --- | --- | --- | --- | --- | --- |
| Age (years) | 239 | 67.1 ± 7.6 | 66.6 ± 6.2 | 0.42 | 0.08 | 0.673 | | 0.55 | -3.13 2.02 | |
| Body Mass Index (BMI)(kg/m^2^) | 237 | 25.7 ± 3.2 | 27.2 ± 3.7 | 2.00 | -0.41 | 0.046* | | 1.50 | 0.03 3.00 | |
| Systolic pressure at baseline (mmHg) | 236 | 126.1 ± 17.3 | 130.5 ± 62.6 | 0.36 | -0.07 | 0.719 | 4.34 | | -19.53 28.26 | |
| Diastolic pressure at baseline (mmHg) | 236 | 75.9 ± 8.6 | 77.5 ± 64.8 | 0.13 | -0.03 | 0.894 | | 1.66 | -23.00 26.31 | |
| Mean Arterial Pressure (mmHg) | 236 | 92.6 ± 9.8 | 95.2 ± 63.7 | 0.21 | -0.04 | 0.833 | | 2.60 | -21.62 26.81 | |
| APOE ε4 risk (presence of at least one ε4 allele) | / | 6:19 | 65:161 | / | / | / | | / | / | |
| In person track sessions attended (range, mean) | 226 | 22-78, 54.0 ± 12.8 | 1-111, 51.2 ± 14.1 | -0.98 | 0.21 | 0.538 | | -1.76 | -8.44 2.85 | |
| Make-up logbook sessions (range, mean) | 157 | 0-39, 10.1 ± 10.5 | 0-80, 12.7 ± 9.7 | 1.22 | -0.26 | 0.225 | | 2.52 | -1.57 6.62 | |
| Total Number of sessions completed (range, mean) | 226 | 22-105, 64.2 ± 15.7 | 1-117, 59.6 ± 17.9 | -1.27 | 0.20 | 0.320 | | -3.58 | -11.76 2.53 | |
| Years of Education Completed | 262 | 16.2 ± 2.5 | 15.9 ± 2.5 | 0.48 | 0.08 | 0.629 | | -0.26 | -1.33 0.80 | |

Supplementary Table 1. Demographics of the MRI sub-study sample compared with the larger Brain in Motion Sample

*Note:* SD = standard deviation

Supplementary Table 2. Sex and Age of participants who did not attend more than 85% of the exercise sessions

| Sex | Age (years) | Percent of sessions attended |
| --- | --- | --- |
| Female | 81.7 | 81% |
| Female | 57.8 | 31% |
| Female | 62.8 | 39% |
| Female | 58.1 | 78% |
| Female | 68.2 | 74% |
| Male | 59.7 | 79% |
| Male | 65.4 | 65% |
| Male | 74.8 | 72% |

Supplementary Table 3. Sex Differences in Blood Pressure and Maximal Aerobic Capacity, Pre and Post intervention, Independent *t*-Test Results

|  |  | Pre-Intervention |  |  |  | Post-Intervention |  |  |  |
| --- | --- | --- | --- | --- | --- | --- | --- | --- | --- |
|  |  | Mean ± SD | *t* | *Cohen’s d* | *p*-value | Mean ± SD | *t* | *Cohen’s d* | *p*-value |
| Diastolic Blood Pressure (mmHg) | Females | 71.3 ± 6.3 | -2.77 | 1.09 | 0.011 | 70.8 ± 9.5 | -1.40 | 0.542 | 0.174 |
|  | Males | 79.5 ± 8.6 |  |  |  | 76.0 ± 9.8 |  |  |  |
| Systolic Blood Pressure  (mmHg) | Females | 122.6 ± 20.1 | -0.94 | 0.36 | 0.359 | 119.9 ± 20.0 | -1.34 | 0.512 | 0.219 |
|  | Males | 128.9 ± 14.4 |  |  |  | 129.3 ± 16.3 |  |  |  |
| Mean Arterial Pressure | Females | 87.8 ± 10.4 | -2.59 | -0.71 | 0.012 | 87.2 ± 11.0 | -1.55 | -0.60 | 0.067 |
|  | Males | 94.9 ± 9.7 |  |  |  | 93.8 ± 11.1 |  |  |  |
| Body Mass Index (BMI) | Females  Males | 23.70 ± 2.0  27.30 ± 3.1 | -3.49 | -1.35 | 0.002 | 23.52 ± 2.1  26.89 ± 3.1 | -3.21 | -1.24 | 0.004 |
| $\dot{V}$O_2_peak (ml/kg/min) | Females | 25.8 ± 6.4 | -0.59 | 0.23 | 0.560 | 26.7 ± 6.4 | -1.17 | 0.427 | 0.255 |
|  | Males | 27.1 ± 4.8 |  |  |  | 29.4 ± 5.7 |  |  |  |
| pp$\dot{V}$O_2_peak (%) | Females | 120.6 ± 22.2 | 2.79 | 0.97 | 0.010 | 126.2 ± 21.1 | 2.01 | 0.711 | 0.055 |
|  | Males | 95.3 ± 29.3 |  |  |  | 111.7 ± 19.4 |  |  |  |
| Complete Years of Education | Females  Males | 15.4 ± 2.0  16.78 ± 2.7 | -1.44 | -0.56 | 0.162 |  |  |  |  |
| Exercise Duration (minutes per week) | Females | 158.4 ± 51.4 | 0.62 | -0.25 | 0.850 |  |  |  |  |
|  | Males | 169.1 ± 33.5 |  |  |  |  |  |  |  |
| Percent Heart Rate Reserve | Females | 54.6 ± 7.2 | 0.02 | -0.07 | 0.847 |  |  |  |  |
|  | Males | 55.1 ± 6.9 |  |  |  |  |  |  |  |
| Resting Heart Rate | Females | 68.5 ± 10.6 | -0.20 | -0.09 | 0.841 |  |  |  |  |
|  | Males | 67.8 ± 7.3 |  |  |  |  |  |  |  |
| Hematocrit (%) | Females | 42.5 ± 2.8 | -5.27 | 2.73 | 0.000024 |  |  |  |  |
|  | Males | 48.4 ± 2.7 |  |  |  |  |  |  |  |

*Note*: Values are mean ± standard deviation

Supplementary Table 4. Age Corrected Cognitive Test Scores Means and Standard Deviation

| Test | Pre Intervention | Post Intervention | *t*-test | Cohen’s *d* | *p*-value |
| --- | --- | --- | --- | --- | --- |
| Letter Fluency | 11.5 ± 2.7 | 12.3 ± 2.2 | -1.89 | -0.32 | 0.070 |
| Categorical fluency | 12.4 ± 2.6 | 12.6 ± 2.5 | -0.40 | -0.09 | 0.692 |
| Categorical Switching | 11.8 ± 3.1 | 11.9 ± 3.2 | -0.05 | -0.01 | 0.963 |
| Delayed recall | -0.6 ± 1.6 | 0.0 ± 1.1 | -3.06 | -0.52 | 0.005* |

*Note*: * indicates significance

Supplementary Table 5. Correlation Between Change in Verbal Fluency, and Verbal Memory Scores to Region of Interest Cerebral Blood Flow

| Region | Letter Fluency Change | | Categorical Fluency Change | | Categorical Switching Change | | Delayed Recall Change | |
| --- | --- | --- | --- | --- | --- | --- | --- | --- |
|  | *r* | *p*-value | *r* | *p*-value | *r* | *p*-value | *r* | *p*-value |
|  |  |  |  |  |  |  |  |  |
| Right Insula Change | -0.35 | 0.092 | -0.15 | 0.482 | -0.29 | 0.170 | 0.14 | 0.523 |
| Left Insula Change | -0.45 | 0.028* | -0.11 | 0.596 | -0.29 | 0.176 | 0.09 | 0.680 |
| Right hippocampus Change | -0.29 | 0.173 | 0.00 | 0.999 | -0.33 | 0.121 | 0.05 | 0.825 |
| Left hippocampus Change | -0.35 | 0.093 | 0.00 | 0.966 | -0.16 | 0.455 | -0.02 | 0.920 |
| Left Pars Triangularis Change | -0.24 | 0.268 | 0.05 | 0.819 | -0.15 | 0.486 | 0.01 | 0.953 |

*Note*: * indicates significance

Supplementary Table 6. Correlation Between Change in Verbal Fluency, and Verbal Memory Scores to Region of Interest Cerebrovascular Reactivity

| Region | Letter Fluency Change | | Categorical Fluency Change | | Categorical Switching Change | | Delayed Recall Change | |
| --- | --- | --- | --- | --- | --- | --- | --- | --- |
|  | *r* | *p*-value | *r* | *p*-value | *r* | *p*-value | *r* | *p*-value |
|  |  |  |  |  |  |  |  |  |
| Right Insula Change | 0.17 | 0.449 | -0.30 | 0.893 | -0.01 | 0.969 | 0.20 | 0.367 |
| Left Insula Change | 0.25 | 0.255 | 0.11 | 0.612 | 0.12 | 0.590 | 0.16 | 0.477 |
| Right hippocampus Change | 0.04 | 0.875 | 0.04 | 0.842 | 0.01 | 0.958 | -0.07 | 0.753 |
| Left hippocampus Change | -0.05 | 0.825 | -0.08 | 0.725 | 0.00 | 0.996 | -0.04 | 0.872 |
| Left Pars Triangularis Change | -0.17 | 0.448 | -0.09 | 0.677 | 0.16 | 0.462 | -0.20 | 0.359 |

*Note*: * indicates significance
